# Chickens can durably clear *herpesvirus* vaccine infection in feathers while still carrying vaccine-induced antibodies

**DOI:** 10.1186/s13567-020-00749-1

**Published:** 2020-02-24

**Authors:** Sylvie Rémy, Gilles Le Pape, David Gourichon, Yannick Gardin, Caroline Denesvre

**Affiliations:** 1 Laboratoire de Biologie des Virus Aviaires, ISP, INRAE, Université Tours, Nouzilly, France; 2 PEAT, INRAE, Nouzilly, France; 3 Anastats, 14 rue de la Bretonnerie, 37000 Tours, France; 4 Ceva Santé Animale, Libourne, France

## Abstract

Marek’s disease (MD) is a major disease of chickens induced by Marek’s disease virus (MDV) associated to lethal lymphomas. Current MD vaccines protect against lymphomas, but fail to prevent infection and shedding. The control of MDV shedding is crucial in order to eradicate this highly contagious virus. Like pathogenic MDV, MD vaccines infect the feather follicles of the skin before being shed into the environment. MD vaccines constitute excellent models to study virus interaction with feathers, the unique excretion source of these viruses. Herein we studied the viral persistence in feathers of a MD vaccine, the recombinant turkey herpesvirus (rHVT-ND). We report that most of the birds showed a persistent HVT infection of feathers over 41 weeks with moderate viral loads. Interestingly, 20% of the birds were identified as low HVT producers, among which six birds cleared the infection. Indeed, after week 14–26, these birds named controllers had undetectable HVT DNA in their feathers through week 41. All vaccinated birds developed antibodies to NDV, which lasted until week 41 in 95% of the birds, including the controllers. No correlation was found between HVT loads in feathers and NDV antibody titers over time. Interestingly, no HVT DNA was detected in the spleens of four controllers. This is the first description of chickens that durably cleared MD vaccine infection of feathers suggesting that control of *Mardivirus* shedding is achievable by the host.

## Introduction

The *Gallid herpesvirus type 2* (GaHV-2), commonly named Marek’s disease virus (MDV) is a highly oncogenic *Alphaherpesvirus* and type-species of the *Mardivirus* genus that causes Marek’s disease (MD) in chickens. This disease is characterized by a number of clinical manifestations including acute death, immunosuppression, and notably deadly lymphoma formation. MD is controlled worldwide exclusively by the combination of host genetic selection, biosecurity and vaccination with live viruses [1]. Several types of vaccine viruses are commonly used against MD, alone or in combination, the two most widely used being the attenuated GaHV2 CVI988/Rispens strain and the avirulent Meleagrid herpesvirus (MeHV) also named turkey herpesvirus (HVT) [1, 2]. HVT is also used as a vector to develop recombinant vaccines against other major viral chicken diseases like the infectious bursa disease (IBD), Newcastle disease (ND) or avian influenza [3–8]. MD vaccines are considered imperfect because they neither stop infection nor shedding of pathogenic MDV from feathers [9–12]. Because MDV remains infectious for weeks in the litters [13], pathogenic viruses can accumulate in the environment of vaccinated birds [12]. Despite the undeniable benefit of current vaccines for chicken health and welfare, the inability of these vaccines to protect from infection and shedding explains the endemic feature of this infection and the difficulty in eradicating it. Furthermore, these characteristics are also suspected to favor the increase of MDV virulence over time [10, 14, 15].

The unique known source of MDV excretion and horizontal transmission is the feather follicle, a complex tissue that generates and accommodates each feather of the skin (reviewed in [1, 16, 17]. MDV reaches feather follicles during the first week of infection and replicates in its epithelium before being excreted. Quantification of viral genome copy number by real-time PCR (qPCR) indicates that MDV replication increases steadily from 1 to 2–3 weeks post-infection and remains stable from that time until the death of the birds [18] (Pasdeloup, unpublished data). Like pathogenic viruses, MD vaccines also reach, replicate, and persist in feathers [19–24]. DNA of MDV and MD vaccines are found in dander and dust indicating a shedding of these viruses [9, 12, 21, 25, 26]. Recently, we showed the presence of protected HVT DNA on the skin surface of vaccinated healthy adult breeders by high-throughput sequencing, suggesting that HVT vaccine is still shed 5 months post-vaccination [27]. To date, no chicken individual or line was reported not replicating or shedding MDV or MD vaccines after being infected or vaccinated. Host genetics is well known to influence the susceptibility or resistance of chickens to develop MDV-induced tumors [1, 28, 29]. In contrast, the influence of the hosts’ genetics on the capacity of chickens to control the replication and shedding of MDV or MD vaccines has been poorly studied and is generally considered as low. Birds from the MD-resistant N2a line had tenfold reduced MDV loads in feathers than birds from the MD-sensitive P2a line at 21 days post-infection [30].

In this study, we addressed two questions: can MDV and MD vaccines be considered as lifelong persistent viruses in chickens of all lines? And can MDV and MD vaccine infections be controlled by the host or even eradicated? The answers to these questions will further our understanding of how to control MD excretion from feathers either through genetics and/or new vaccination approaches. To answer these questions, a recombinant HVT vaccine carrying the F gene of Newcastle Disease virus (rHVT-ND) was used as a model of *Mardivirus* because (i) HVT is non-pathogenic for chickens and preserves birds’ welfare, (ii) HVT infection can be monitored throughout the animal life and (iii) HVT-ND uptake is traceable thanks to the F gene, inducing a high and long-lasting antibody response to the NDV F-protein after a single injection [31]. We found for the first time chickens that durably cleared MD vaccine infection of feathers suggesting that control of *Mardivirus* shedding is achievable by the host.

## Materials and methods

### Experimental chickens and in vivo study design

Forty birds were used for this experiment, with four groups of ten female birds, one group for each chicken line: Fayoumi (Fa), Nunukan (Nu), a broiler grand-parental line, named pH+ or “ultimate” (herein pH) [32] and a commercial brown egg layer line (He). The Fayoumi line was chosen because of its relative resistance to several infectious diseases, like coccidiosis (Eimeria) and Newcastle disease [33, 34]. The Nunukan line was chosen because of its very slow feathering phenotype due to the sex-linked dominant K^n^ allele [35] resulting in little feathers at hatch and very slow growing primary feathers. The pH and He line were chosen as representative of commercial broilers and layers, respectively. Fa, Nu and He birds come from our birds’ facility (PEAT) and He were obtained from Hendrix Genetics as fertilized eggs. All chicks were hatched in our facility. Birds were reared indoors on the ground, all together in one experimental bird house, from hatch until 18 weeks. After that, birds were reared in cages until the end of the experiment as in breeding program. All chicks harbored maternally derived antibodies, including against MDV and NDV. One-day-old chicks were vaccinated subcutaneously against Marek’s disease (MD) and Newcastle disease (ND) with a single injection of deep-frozen cell-associated rHVT-ND recombinant vaccine (Vectormune^®^ ND, CEVA, batch372-4213, 5028 pfu in 0.2 mL of vaccine diluent per chick) using an Avijector. Birds were also vaccinated against others infectious diseases as grandparent breeders according to the vaccination protocol of our animal facility as previously reported [27]. Birds were euthanized at 41 weeks of age according to EU rules (directive 2010/63/EU) and necropsied.

Vaccinated chickens were sampled for 3 to 4 growing feathers at 14 days post-vaccination (from the axillary tracts, the neck and/or the rump) and every 3 weeks after that, until week 41 of age, resulting in 14 feathers samples per bird. Peripheral blood was collected at 9 time-points (2, 5, 8, 11, 17, 24, 29, 35 and 41 weeks) for serum preparation and titration of antibodies to NDV. At necropsy (week 41), spleens were harvested for HVT load quantification. Experimental procedure was carried out according to the French guidelines (protocol APAFIS n°#3712-2016012116461712v2).

### DNA preparation and absolute quantitation of HVT load by qPCR in feathers and spleen

Feathers tips material was collected mechanically on a small piece of Whatman paper, by pressing and rubbing a scalpel blade on the sheaths of growing feathers. Feather pulp and elements from the feather soft shaft were subsequently chopped and their DNA extracted. For that, the Whatman paper was soaked in 220 μL of Proteinase K/ATL lysis buffer overnight at 56 °C and extracted with the QIAamp DNA Mini kit (Qiagen), according to the manufacturer protocol. For the spleens, DNA was extracted from about 25 mg of tissue as previously reported [36]. Quantification of HVT genome copy number was performed using the TaqMan technology. HVT and iNos primers and probes were previously described respectively [22, 37], and purchased from Eurogentec. Both probes were labeled with FAM-BHQ1. Each qPCR mixture contained (i) 10.5 μL of 2 X GoTaq Probe qPCR master mix (Promega) including 0.9 µM of each gene-specific primer and 0.25 µM of the gene-specific probe (ii) mixed with 250 ng of DNA in 9.5 μL. Both genes were quantified independently in triplicate. The standard curve for HVT was obtained by performing qPCR on a serial tenfold dilution of a bacterial artificial chromosome (BAC) containing the entire HVT genome starting at 47.5 pg (corresponding to 2.7 × 10^5^ copies) (the HVT BAC [38] was a kind gift from Prof. V. Nair, The Pirbirght Institute, UK). The standard curve for iNos was performed in the same manner, starting from 47.5 pg (5.68 × 10^6^ copies) of a pBS iNos plasmid. The positive cut-off points correspond to ≥ 27 and 57 copies of HVT DNA and iNos, respectively, according to the standard curves. All qPCRs were performed in a CFX96 RealTime System (BioRad, Marnes-la-Coquette, France) and the results were analyzed using the CFX manager software (version 3.1) (BioRad). For each sample, HVT load corresponded to the number of HVT genome copy number per 10^6^ cells (meaning per 2 × 10^6^ iNos copies). Data for a triplicate were ruled to be non-interpretable, when at least two Cq of a replicate showed variations greater than 0.5 or when only one out of the three Cq was obtained, and that on two independent HVT qPCRs using the same DNA sample. Note that for iNos qPCR, all the results were interpretable.

### NDV antibody titrations

To detect antibodies to NDV in the serum samples, both ELISA (ID screen Newcastle Disease indirect kit, IDvet, France) and hemagglutination inhibition (HI) test were used. HI test was performed using standard method against 4 HAU of La Sota antigen and titers are given in Log_2_. Positivity limit of HI and ELISA titer was ≥ 2 Log_2_ as previously described [31] and ≥ 993, respectively.

### Statistical analysis

For HVT loads in feathers, 46 data points were initially missing due to the lack of growing feathers or uninterpretable triplicates. After re-imputations for two data (using polynomial curve fitting), 44 data points were still missing, all from week 24. For antibody titers, only one data point was missing and re-imputed.

Because of low sample sizes (10 independent subjects in each line) only non-parametric tests were used. Permutation tests were used on numerical values when homogeneity of variances was verified, otherwise on ranks. Comparisons of proportions were performed by the Fisher exact test. For comparisons of independent samples (e.g. lines at a given date) or dependent samples (e.g. dates for a given line), permutation methods for analysis of variance (ANOVA) were used. For ANOVA in case of mixed models (e.g. several lines at different dates), a non-parametric ANOVA-like test using ranks and adjusted *p*-values for pairwise comparisons was used [39, 40]. The principal component analysis (PCA) used three active variables: HVT load, anti-NDV Ab titer by HI, and anti-NDV Ab titer by ELISA. Note that the chicken line was not a variable, but a supplemental element (“illustrative variable”). In PCA, we used only the weeks at which both Ab titers and HVT loads were measured and the subjects for which HVT loads were not missing. Here, each line is an individual measure and not a subject. The R software version 3.4.3. was used for computing and plots [41].

## Results

We evaluated the persistence of HVT infection in feathers of four lines (Fayoumi, Fa; an egg layer line, He; Nunukan, Nu; and a parental broiler line, pH) vaccinated with HVT-ND at hatch. For that, we measured HVT loads by qPCR in growing feathers tips of forty birds (ten per line) from week 2 and every three weeks over 41 weeks. On 520 expected measures, 476 were obtained, after two imputations (Additional file 1). The 44 missing data were from week-24 birds, mostly due to the lack of growing feathers, especially in the pH line. The data were complete for 25 birds (Fa: 8, He: 8, Nu: 7, pH:3).

### HVT load in feathers per line, regardless of the time and over time

**Figure 1:**
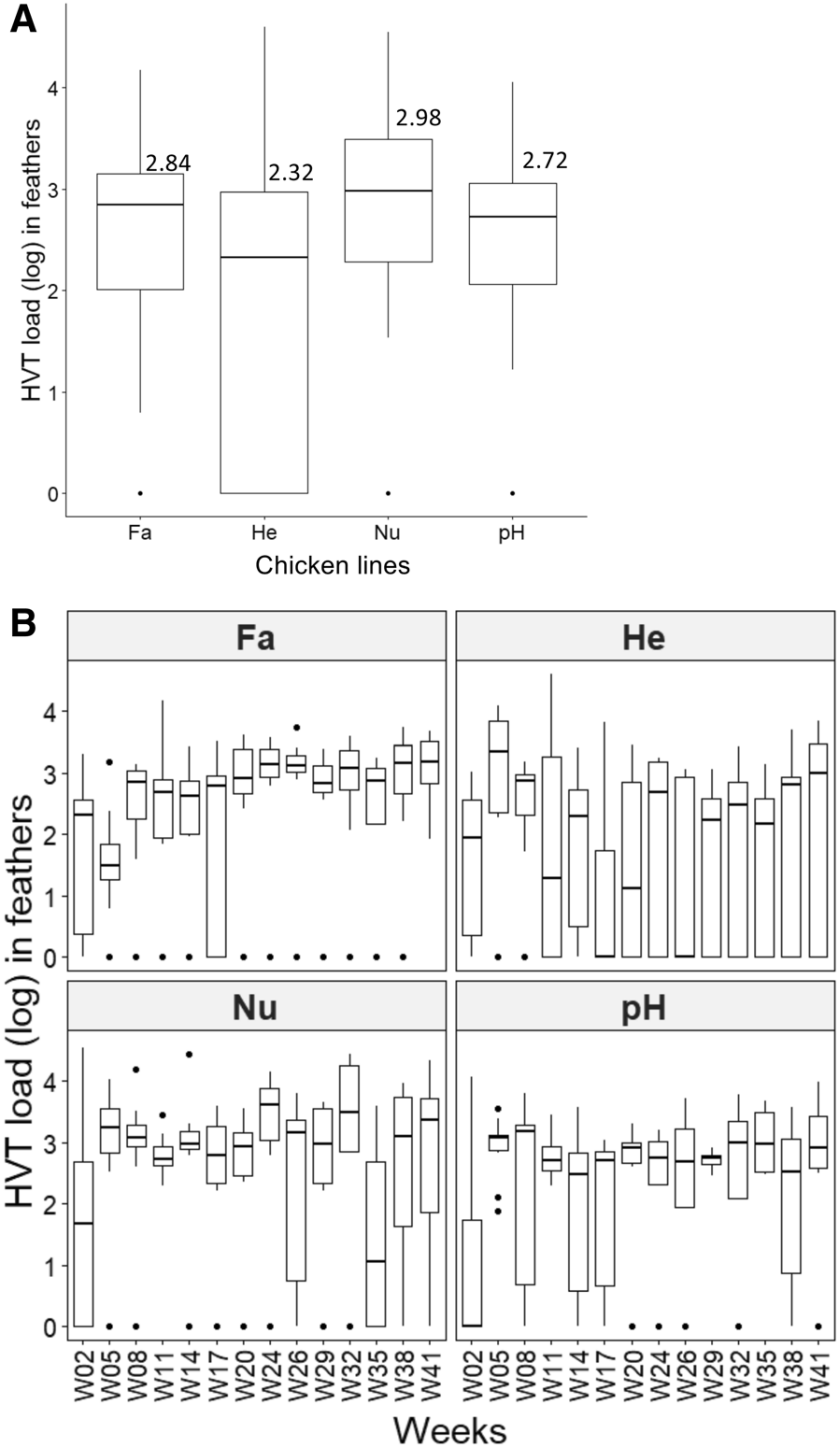
**Influence of the chicken line on HVT load in feathers.** Each HVT load corresponds to the log_10_ of HVT genomes number per million cells. Data are shown in Tukey boxes with the medians shown as thick horizontal bars. **A** HVT load per line regardless of the time. The medians (log) are indicated above each box. HVT loads were of moderate level in the four lines and not significantly different; **B** HVT load per line over time. He and Fa showed significant differences of HVT loads in feathers over time, whereas Nu and pH did not.

HVT loads in feathers were of a moderate level for the four lines, with medians of 692 for Fa, 210 for He, 952 for Nu, and 530 for pH (Figure 1A). A non-parametric ANOVA-like test for mixed models (nparLD) indicated that regardless of the dates, the differences in HVT loads in feathers between lines were not significant (*p* = 0.1297).

We next examined HVT load per line over time. A non-parametric ANOVA-like test (nparLD) indicated that changes in HVT loads over time were significant (*p* < 0.001) (not shown). Separate comparisons of changes over time were performed for each line and indicated that the changes of HVT loads over time were significant only for two lines, Fa (*p* < 0.001) and He (*p* = 0.044) lines. For the Fa line, the HVT loads remained high with a low variability (Figure 1B). For the He line, although the HVT load medians remained high, several birds had zero HVT load after week 11 (Figure 1B). Although all lines globally produce similar and moderate amounts of HVT DNA in feathers regardless of the time, differences were observed between lines over time.

### Dynamic of HVT load in feathers per subject over time

**Figure 2:**
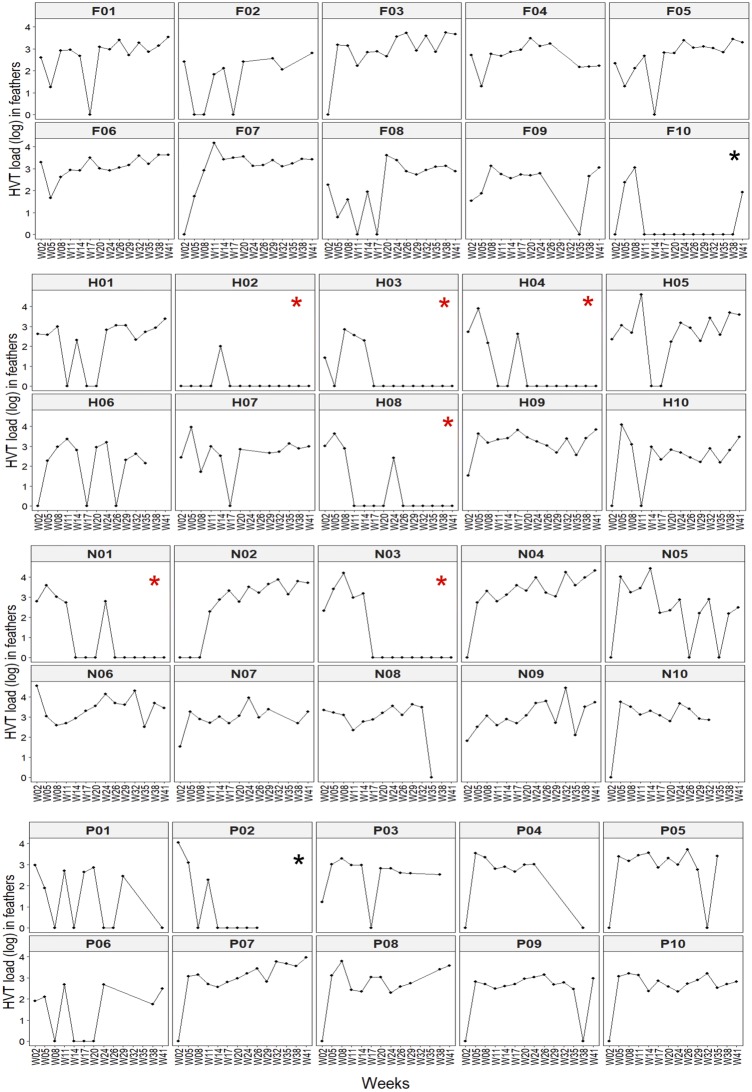
**Dynamic of HVT load in feathers per subject over time.** Each HVT load corresponds to the log_10_ of HVT genomes number per million cells. Each dot corresponds to the HVT load at a time point. When the dot is missing, the datum was missing. Missing data were numerous for the pH birds after week 24, due to the lack of growing feathers. The birds exhibited two major profiles: (i) persistent high loads or variable loads with positive loads interspersed with null loads (e.g. Fa04 and Fa08, respectively); (ii) low producer profile including controllers (indicated by a red star), with several successive zero loads notably from week 14 and including at week 41 (e.g. He03 and Nu03*)*. The low producers but not controllers are marked with a black star (*), like Fa10 due to its positivity at week 41.

HVT load in feathers was subsequently analyzed per subject in each chicken line over time (Figure 2). We looked at early time points like in previous reports and found that 24% of the 120 feather samples had a negative HVT load from week 2 to 8. However, among the 40 birds, only three birds (7.5%; Fa02, He02, and Nu02) had 2 successive negative feather samplings during that period (Figure 2). During the 41 weeks of the experiment, two major profiles were identified in all the lines. The first profile corresponded to birds with high (e.g. Fa04, Nu07) or variable (e.g. Fa08, Nu05, He06) HVT load over time. This profile was the most common, corresponding to 32 birds (80%): nine birds in Fa, six birds in He, eight in Nu, and nine in pH. Six birds had HVT DNA detectable in all their feather samples over 41 weeks (2Fa, 1He, 3Nu), and 12 birds had only one null sample (4 Fa, 1 He, 3 Nu, 4 pH), representing together 45% of the birds. Note that in birds with variable HVT load, the low loads were not systematically at later time points (e.g. Fa08, Nu02). The second profile corresponded to birds showing no HVT DNA in feathers for several weeks after one or several positive measures. Eight birds (20%) belonged to this group: one bird in Fa (Fa10), four birds in He (He02, He03, He04, He08), two birds in Nu (Nu01, Nu03), and one bird in pH (pH02). We called these birds “low producers”. In this group, HVT DNA became undetectable starting from week 11: week 11 (Fa10), week 14 (pH02), week 17 (He02, He03, Nu03), week 20 (He04), and week 26 (He08, Nu01). In low producers, we observed peculiar patterns of HVT detection. For the Fa10 bird, HVT DNA re-appeared in feathers at week 41 after being undetectable in 10 successive measures during 33 weeks. He02 showed only one positive HVT feathers sample which was ascertained on two independent DNA extractions of the same harvest, eliminating sample contamination at the first extraction. The status of pH02 as a low producer is uncertain due to the lack of the five last samples. Six birds (He02, He03, He04, He08, Nu01, Nu03) in the low producers’ group were negative for at least six consecutive feathers sampling, including the latest one at week 41. They were named “controllers”. In conclusion, a few controllers from two lines durably cleared HVT infection in feathers, while most birds showed a persistent infection over 41 weeks. The clearance of HVT infection in feathers was observed late post-vaccination, after 14 weeks.

### No super-producers were identified from HVT feathers loads

**Figure 3:**
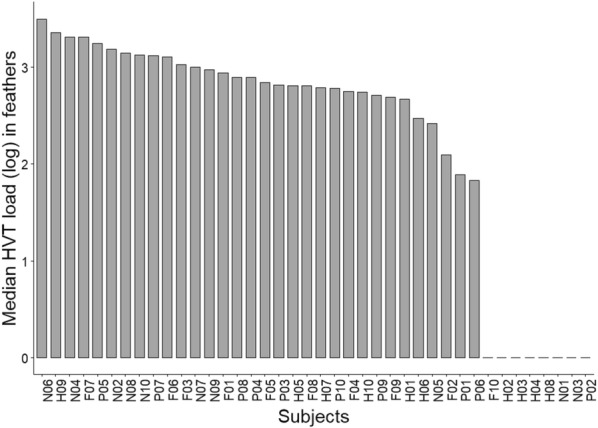
**Ranking of subjects according to HVT load in feathers.** The graph is based on HVT loads median (log) per bird. The medians were ranked from the highest to the lowest (left to right). This graph highlights two categories of birds, high producers and low producers with a null median, 20% of birds being in that last category. No super-producers were identified among the high producers.

We next looked for the presence of super producers. For that, the median of HVT loads (log) for all dates was calculated for each bird and ranked from the highest to the lowest median (Figure 3). The eight low producers identified above showed a zero median, as expected, and clearly clustered in a group. For the other birds, the medians (log) ranged between 3.5 and 1.83, with birds from the four lines being interspersed. No obvious break in the bars’ heights was observed, indicating that this second group with a positive median is quite homogenous and devoid of super producers.

### Antibody titers to NDV by hemagglutination inhibition (HI) and ELISA in each line over time

**Figure 4:**
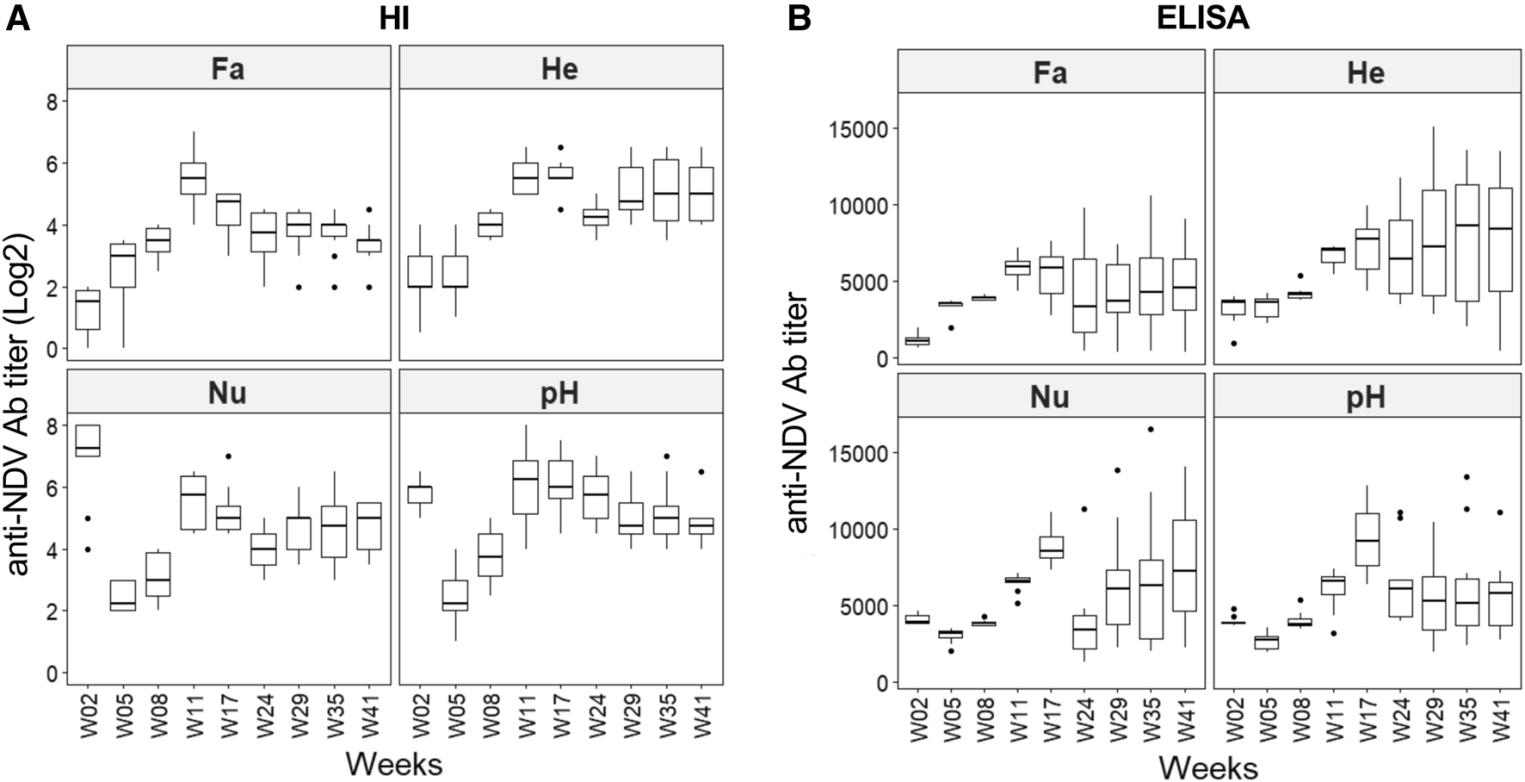
**Evolution of serum antibody (Ab) titers to NDV per line.** Data are shown in Tukey boxes with medians shown as thick horizontal bars. **A** Anti-NDV antibody titers by HI (in log_2_) per week and per line. **B** Anti-NDV antibody titers by ELISA per week and per line. rHVT-ND vaccination induced high and long-lasting antibody titers to NDV in the four lines, according to both titration methods. Antibody titers peaked at week 11 by HI and mostly at week 17 by ELISA.

Serum antibodies to NDV were titrated by HI and ELISA tests in order to evaluate maternally derived antibodies levels as well as the development and duration of the antibody response induced by rHVT-ND vaccination. No data were missing from the 720 expected data points, after a single imputation for lack of sample (H09/W02) (Additional file 1). We examined ND antibody titers in each line over time with both methods (Figure 4). The four lines had significantly different levels of maternally derived anti-NDV antibodies (statistical analysis not shown): negative by HI (≤ 2) to low by ELISA (1115) for Fa, low by HI (2) and moderate by ELISA (3631) for He, and high by HI (≥ 6) or moderate by ELISA (about 3880) for Nu and pH. At week 5, a decrease in maternally derived antibody titers was observed in Nu and pH lines. In contrast, antibodies remained constant or started to rise in He and Fa lines, respectively, which is coherent with their low titers at week 2. At week 8, antibody titers started to increase in all lines, with both methods, regardless of the maternally derived antibody titers. The antibodies titers peaked at week 11 in all lines with HI (between 5.5 and 6.25) and between week 11 and 17 with ELISA (between 6000 and 9150). From week 11, the titers by HI slightly decreased but remained above 3.5 until week 41. The variability of HI titers was similar over time from week 5 and that in each line. In contrast, with the ELISA method, the variability tended to increase after week 17, even if the medians remained elevated for all lines (> 6000). The ELISA results showed that antibody titer medians tended to rebound, after a decrease at week 24. In conclusion, our HI and ELISA results showed that rHVT-ND vaccination induced high and long-lasting serum antibody titers to NDV up to 41 weeks in all four chicken lines. In addition, the highest serum antibody titers to NDV were observed at week 11 to 17 in all lines, regardless of maternal antibodies titers.

### Antibody titers to NDV by HI and ELISA in each subject over time

**Figure 5:**
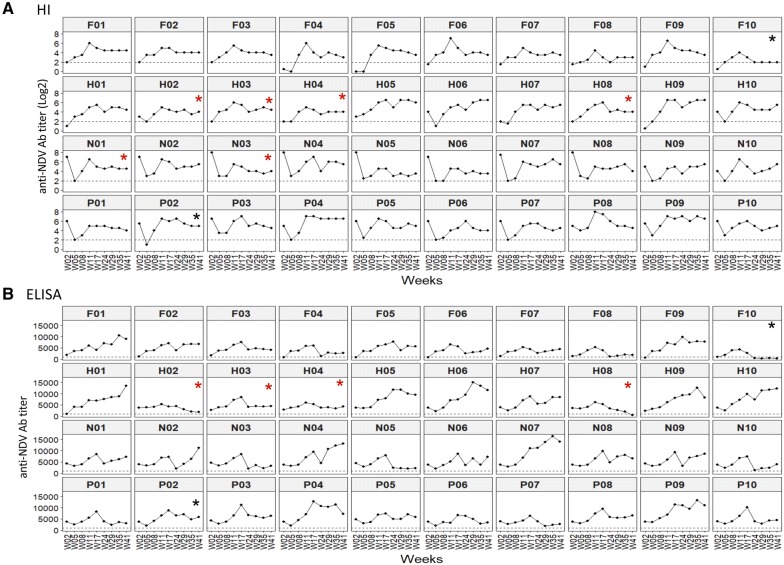
**Dynamic of anti-NDV antibody titers per subject over time.** Each dot corresponds to the Ab titer at a time point. The controllers are highlighted by a red star (*) and the low producers not controllers by a black star (*). **A** Antibody titers to NDV by HI (in log_2_) per bird. The horizontal dotted lines show the threshold of the assays set at 2. **B** Antibody titers to NDV by ELISA per bird. Ninety-five percent of the birds exhibit positive antibody titers to NDV by HI and ELISA from week 8 and until week 41 post-vaccination.

NDV antibody titers measured by both methods were subsequently analyzed by subject over time (Figure 5). With the HI test (Figure 5A), we observed 29 birds (72.5%) with a negative titer (≤ 2) at weeks 2 and/or 5. These negative titers corresponded to birds with no maternally derived antibodies (He and Fa birds) or after a decrease in these antibodies (especially in pH and Nu birds). From week 8, all birds exhibited a titer superior to 2 log_2_ with the exception of Fa10 (see below). With ELISA at early time points, antibody titers were positive (above 1000), except for five birds (Fa04, Fa05, Fa06, Fa09, He01) (Figure 5B). Most birds had titers that ranged from 2500 to 10^4^. We did not observe birds with high titers in the Fa line. At week 41, all birds still harbored ELISA titrated antibodies in the range of 1939 to 14 011, except two which were at the threshold level (He08 and Fa10). According to both titration methods, all birds from the four lines developed antibodies to NDV after vaccination and these antibodies persisted for 41 weeks in 95% of the birds.

We paid special attention to the antibodies in the six controller birds (Nu01, Nu03, He02, He03, He04, He08) in order to examine whether the lasting absence of HVT DNA infection in feathers starting at 4 to 6 months post-vaccination was associated with lower antibody titers or not. At week 41, all birds still exhibited positive titers by HI (of 4 or 4.5). By ELISA, the six controllers had positive titers (Nu01: 7193, Nu03: 3168, He02: 1951, He03: 4406, He04: 4356) except one (H08: 449). The low producer Fa10 bird was the only bird with negative antibody titers by HI and ELISA from week 24. Note that this bird had only HVT DNA detectable in feathers at three time points (Weeks 5, 8, and 41). In conclusion, 95 percent of the birds, including the controllers, exhibited positive antibody titers to NDV by HI and/or ELISA through week 41 post-vaccination.

### No relationship exists between HVT loads in feathers and antibody titers to NDV

**Figure 6:**
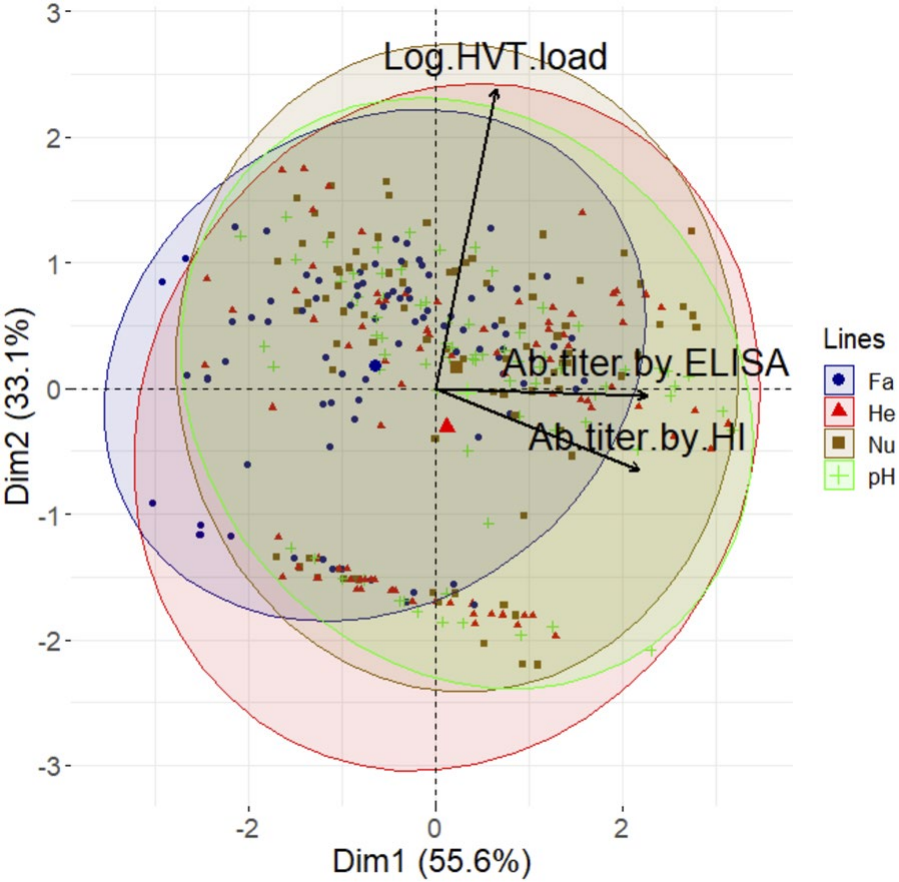
**Relationships between HVT load in feathers and serum antibody titers through a PCA.** PCA factorial plot for which three variables used were: HVT load in feathers, anti-NDV Ab titer by HI and Ab titer by ELISA. This plot is highlighting the chicken line groups. All individuals at all weeks, for which the data were completed were plotted. The barycenter of each chicken line group is represented by an enlarged and filled forms: blue dot (Fa), red triangle (He), brown square (Nu), and green cross (pH).

To examine the relationship between HVT loads in feathers and antibody titers to NDV by the two titration methods, we used a principal component analysis (PCA). Figure 6 shows the three variables of the PCA as well as all individuals. The two first dimensions represented 88.7% of the total variance. This analysis showed that the Nu group (brown ellipse) is located on the top (indicating high HVT loads), and the He group (red ellipse) on the bottom (indicating low HVT loads), as described above. This analysis also clearly demonstrated a correlation between the two antibody titration methods (vectors with a low angle). In contrast, the orthogonality between the HVT load vector and the NDV antibody titer vectors indicated an independence between HVT loads in feathers and antibody titers. Lastly, this analysis also showed a series of aligned dots at the bottom of the graph, indicating very low or zero HVT loads. These dots include data at early time points when the HVT genome was still undetectable, and data from the controllers at later time points. The colored ellipses corresponding to the four chicken lines were superimposed indicating low variations between lines in terms of HVT loads in feathers and ND antibody titers. The only low variation was a slight shift of the Fayoumi’s ellipse and Fayoumi’s group barycenter (thick red dot) towards the left (Figure 6), indicating lower antibody titers, as previously mentioned. In conclusion, no relationship between HVT loads in feathers and antibody titers to NDV by HI and ELISA was observed by PCA, whereas a strong relationship was observed between the two antibody titration methods.

### A relationship exists between HVT load in feathers and spleens

**Figure 7:**
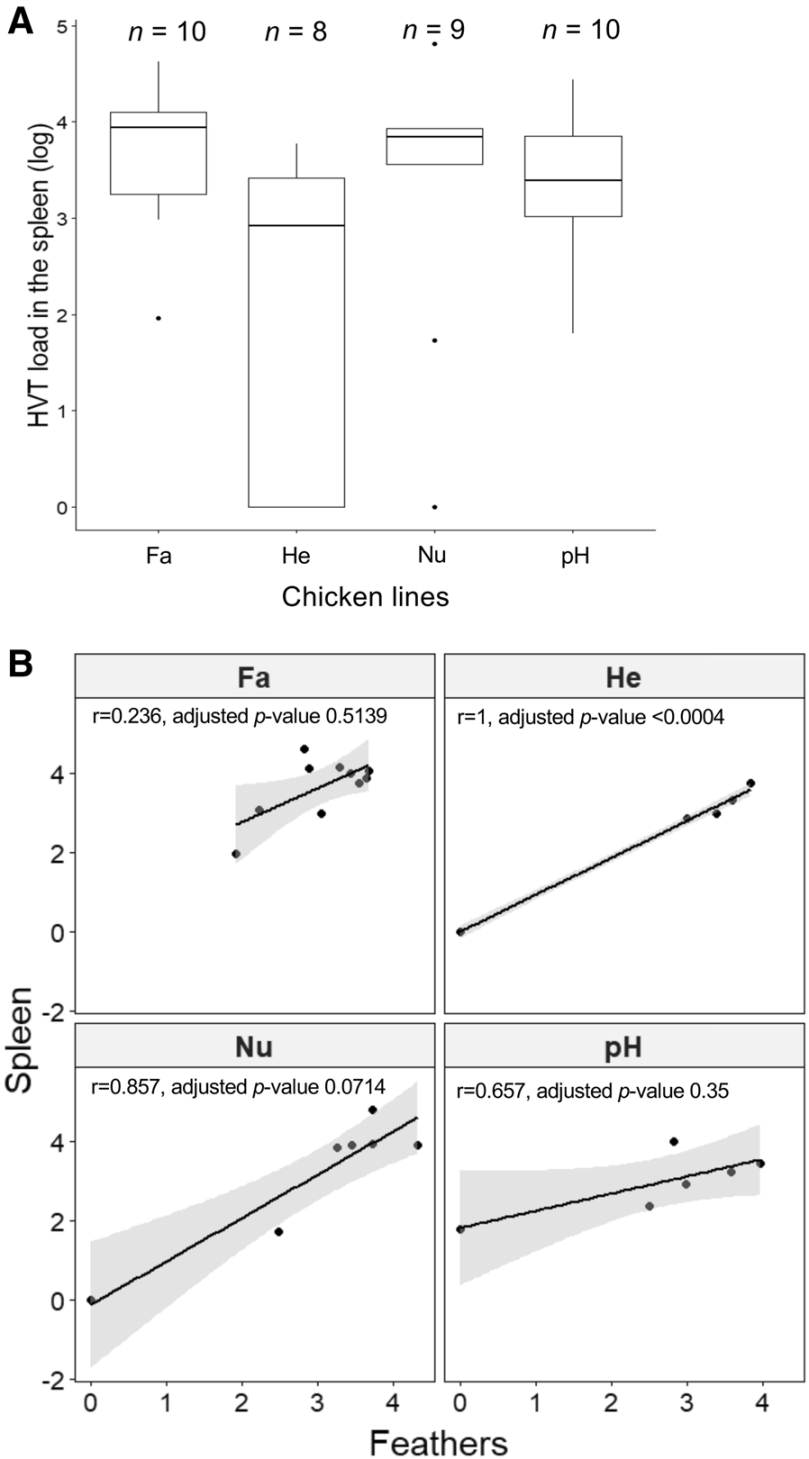
**HVT viral loads in the spleen and its relationship with HVT loads in feathers. A** HVT loads in the spleen. Each HVT load corresponds to the log_10_ of HVT genomes number per million cells. Data are shown in Tukey boxes with the medians shown as horizontal bars. Four birds had null spleen loads: He02, He03, He04, and Nu03. **B** Relationship between HVT loads in spleen and feathers at week 41. A correlation analysis by lines was performed through Spearman test with a Holm’s adjustment of *p*-values. Fa line (*n* = 10), He line (*n* = 7), Nu line (*n* = 7), pH line (*n* = 6). Spearman rho and holm adjusted *p*-values are indicated for each line.

MD vaccine are known to persist for several weeks in the spleen of vaccinated birds [42, 43]. In order to examine whether HVT persistence in feathers was related to its persistence in the spleen, we quantified HVT loads in the spleen of each bird at week 41 by qPCR. Of the 40 data points expected, 3 were missing (Additional file 2). The median HVT loads (log) in the spleen were 3.85, 3.39, 3.94, and 2.92 for Nu, pH, Fa, and He lines, respectively (Figure 7). There were no significant differences in HVT loads in the spleen between the four lines using an ANOVA by permutations (*p* value = 0.0597). Interestingly, four birds had null values (Nu03, He02, He03, He04) and were previously identified as controllers. Interestingly, two birds identified as feathers low producers, but not controllers, had the lowest HVT loads in the spleen (pH01, 64; Fa10, 92). To examine the relationship between HVT loads in the spleen and feathers, a correlation analysis was performed in each line through a Spearman test with a Holm’s adjustment of *p*-values (Figure 7B). A positive correlation was found between the viral loads in these two organs in each line. These results indicate that a low HVT load in the spleen was associated with a low load in the feathers. The correlations were significant only for the He line (adjusted *p* < 0.0004), in which 7 subjects had complete data.

## Discussion

The infection of feathers by HVT was monitored by HVT-specific qPCR allowing an absolute quantification of HVT genomes. Our feathers tips material contained both feather pulp and epithelium. Therefore, we estimated the possible contribution of HVT latently-infected lymphoid cells theoretically present in the pulp. In normal condition, the percentage of CD4+ lymphocytes in the pulp represents about 1% of the cells [44]. Knowing that HVT load in the PBMC of HVT-vaccinated unchallenged birds is about 2 × 10^3^ copies per million cells at 6 weeks post-vaccination (unpublished data), we could expect 20 HVT genomes coming from latently lymphoid cells in 10^6^ cells. This number is very low compared to the viral loads quantified. Therefore, we assumed that HVT genomes detected in feathers tips came mostly from lytically infected epithelial cells. The HVT loads in the feathers for the four lines were moderate, with medians never exceeding 10^4^ genome copies per million cells, even at the early time points. This finding is in the range of HVT loads previously reported at 8 weeks in the feather tips of another chicken line also provided with maternal antibodies [45]. This indicates that HVT loads in vaccinated birds are about 10^3^-fold lower compared to MDV loads in birds vaccinated with CVI988 or infected with MDV virulent strains [19, 45]. The origin of HVT reduced loads compared to MDV remains unclear. Prasad and Spadbrow proposed that HVT would not be able to form enveloped mature virions in the skin [46]. However, a reduced replication ability or defective morphogenesis of HVT in chicken keratinocytes has not yet be proven.

Eighty percent of the birds (32/40) showed a persistent HVT infection in feathers over 41 weeks. This result is coherent with our previous finding that a pool of adult chicken skin swabs contains HVT protected DNA [27]. This result is also coherent with a recent study obtained with chickens vaccinated with CVI988, in which 40 to 95% of the feather samples collected between 60 and 90 days post-vaccination were qPCR positive [47]. In that report, the authors showed that CVI988 DNA was detectable in the dust from week one until week 50, with a peak at weeks 3–6 and a 1000-fold decrease after week 26.

Among these 32 birds, 18 had zero or one negative sample (over the 14 samples harvested) indicating a chronic HVT infection of feathers. This also highlights a complete failure of the immune response to control viral infection at these cutaneous structures. For the remaining 14 birds, the loads were varying, with alternating positive and negative samples over time. Such a profile with irregular negative samples can be interpreted in different ways: (i) The host eliminated the infection from some feathers, which get subsequently re-infected possibly by the arrival of latently infected cells, (ii) part of the feathers of a bird remain negative or below the detection threshold, and/or (iii) a sampling bias. Indeed, a chicken has more than 20 000 feathers [48] and we extracted DNA from only 2–3 feathers at each sampling. In the absence of any systematic analysis of a large population of feathers from several birds vaccinated with HVT, it is impossible to determine whether such sampling was sufficient to ascertain the presence or absence of HVT in feathers. Therefore, we cannot completely eliminate that some HVT negative samples were due to sampling variability. (iv) Lastly, the presence of qPCR inhibitors (i.e. feather pigments) in the DNA of negative samples was not considered as a possible explanation, because all samples were positive with an iNos qPCR.

One of the most important findings in this study is the discovery of six controller birds, which have undetectable HVT DNA in feathers starting between weeks 14 and 26 and up to the end of the experiment at 41 weeks. This finding is of high interest because it shows for the first-time the control and clearance of a mardivirus infection in the feathers. Interestingly, four controllers had also no detectable HVT DNA in their spleen, indicating that either the HVT vaccine was totally eradicated or present at a very low level in both tissues below the threshold of sensitivity of the qPCR.

The origin of the persistent infection in the feather follicles by MDV or HVT observed in most birds remains unknown. Various hypotheses could be proposed: (i) a chronic infection of the feathers associated with an inefficient immune response or (ii) a regular reinfection of the feathers from a viral reservoir located outside the skin, like the spleen. The positive correlation between HVT loads in the spleen and feathers supports the second hypothesis. Further studies are warranted to address this issue more directly.

For human alphaherpesviruses (HHV-1, HHV-2 and HHV-3), infection of the skin or mucosa are usually rapidly controlled by the immune response in immunocompetent individuals and these viruses do not induce a persistent infection of these tissues [49, 50]. For example, after perioral or genital infections with HSV-1 or HSV-2, a rapid clearance occurs within 6 to 12 h in immunocompetent adults [49]. Recently, the role of skin-resident memory CD8+ T cells was discovered as a major player in this process [51]. For MDV and MD vaccine models, the halt of viral infection in feathers has never been shown before. In this study, we demonstrate for the first time that such a halt is possible in a small number of animals. However, we have no evidence of a molecular mechanism. The very late control after several weeks post-infection, suggests that the molecular mechanism involved in HVT clearance is different from the one depicted with HSV viruses. The fact that only a few birds were able to control the infection of feathers supports the role of genetic factor(s) shared by a small number of birds.

Our data indicate that the NDV antibody response induced by HVT-ND vaccination at 1-day-old is long lasting and reach high titers for 95% of the chickens in the four different lines. These results are consistent with our previous report in which we followed a commercial layer line vaccinated similarly up to 72 weeks of age [31]. We also observed a strong correlation between HI and ELISA NDV antibody titration, similarly to that previously reported with SPF White Leghorn chickens (HI test vs. an IgG in-house ELISA) [24]. Although, the two methods measured different types of antibodies and showed different titers, both methods might be used for NDV protection prediction which is based on NDV antibody seropositivity. Interestingly, all controllers showed ND antibodies by HI through week 41, signifying that despite no apparent replication of HVT vaccine in feathers for weeks, the antibody response was long-lasting. By ELISA, the ND antibodies also persisted through week 41, except for one bird. This result suggests that rHVT-ND persistence in feathers is not mandatory for serum NDV antibodies’ persistence over 41 weeks. Although four of these birds had also no detectable HVT genome in the spleen at week 41, we cannot rule out that some HVT replication occurred in the spleen earlier, contributing to the lasting of the antibodies.

Vaccine uptake is an important assessment for bird disease management, especially for endemic infectious diseases in large groups of birds. For rHVT-ND, the vaccine uptake is routinely controlled by two methods: the detection of HVT genome by PCR in feathers or spleen, and the detection of NDV antibody titration. In this study, we did not observe a relationship between HVT loads in feathers and NDV antibody titers when all the samples were considered. This result points out that these two methods are not equivalent for measuring HVT uptake. This result contrasts with one of our collaborator’s results [24]. The differences between our two studies can mainly be attributed to differences in the design of the experiments. In our collaborator’s experiment, a SPF line devoid of maternal antibodies was used and HVT load in feathers was monitored for a short period of time of 6 weeks, with a less sensitive qPCR (not shown). According to our results, in birds harboring maternal antibodies, we recommend measuring NDV antibody titers when the assessment of vaccine uptake is performed after week 8. At earlier time points, the detection of HVT genome in feathers appears a preferable method. However, this method could give rise to false negatives when performed at a unique time point. Indeed, between weeks 2 and 8, we observed 24% of negative HVT loads in feathers, whereas antibody titrations performed at week 11 showed that all these birds were correctly vaccinated.

Several important questions remain: Is a control of a mardivirus exhibiting higher virus loads in feathers than HVT (e.g. CVI988) also possible, as such virus would likely be more representative of a virulent MDV? Why the immune response fails to clear the virus in most birds? What is the molecular basis of the mardivirus infection control in controllers and why does it occur so late? Is there a relationship between HVT control and the host’s genetic background? It would be interesting to follow MDV loads in feathers of HVT-vaccinated birds to explore whether some controllers are also identifiable for HVT and/or MDV.

We addressed the ability of chickens to control mardivirus infection in feathers by using a rHVT-ND vaccine as a mardivirus model. Our data demonstrate that individuals can achieve long-lasting control of rHVT-ND infection in feathers after 14 weeks. This phenotype was observed in at least 2 lines (He, Nu), indicating that control of Mardivirus infection in feathers by the host is feasible. Importantly, our results also demonstrate that birds that control vaccine replication for weeks still have vaccine-induced antibodies and indicate that vaccine replication in feathers appears not mandatory for long-lasting ND protection.

## Supplementary information

**Additional file 1. Raw data of HVT loads in feathers and antibody titers.**

**Additional file 2. HVT loads in spleens at week 41.**

## Data Availability

All raw data (viral loads in feathers and spleens as well as antibody titers) are provided in Additional files 1 and 2.

## Abbreviations

Fa: Fayoumi
HAU: hemagglutination unit
HI: hemagglutination inhibition
HVT: turkey herpesvirus
MD: Marek’s disease
MDV: Marek’s disease virus
ND: Newcastle disease
NDV: Newcastle disease virus
nparLD: non-parametric analysis of longitudinal data
Nu: Nunukan
PCA: principal component analysis
qPCR: real-time PCR
