# Supplementary material for: Chickens can durably clear herpesvirus vaccine infection in feathers while still carrying vaccine-induced antibodies
Source: Vet Res. 2020 Feb 24;51:24. doi: 10.1186/s13567-020-00749-1 (PMC7041111; doi:10.1186/s13567-020-00749-1)
Supplement: Supplementary file 1 — Additional file 1. Raw data of HVT loads in feathers and antibody titers. [file 13567_2020_749_MOESM1_ESM.pdf]

Subject	Line	Date	Group	HVTLog10	AcHILog2	AcELISA
N01	Nu	W02	NuW2	2.805	7	4342
N02	Nu	W02	NuW2	0.000	7	3925
N03	Nu	W02	NuW2	2.326	8	4594
N04	Nu	W02	NuW2	0.000	8	3807
N05	Nu	W02	NuW2	0.000	8	4511
N06	Nu	W02	NuW2	4.541	7	3758
N07	Nu	W02	NuW2	1.535	7.5	3830
N08	Nu	W02	NuW2	3.343	8	3790
N09	Nu	W02	NuW2	1.812	5	4275
N10	Nu	W02	NuW2	0.000	4	3834
P01	pH	W02	pHW2	2.980	6	3824
P02	pH	W02	pHW2	4.053	5.5	3733
P03	pH	W02	pHW2	1.223	6.5	4269
P04	pH	W02	pHW2	0.000	5	3833
P05	pH	W02	pHW2	0.000	6	4783
P06	pH	W02	pHW2	1.908	6	3835
P07	pH	W02	pHW2	0.000	6	3919
P08	pH	W02	pHW2	0.000	5	3814
P09	pH	W02	pHW2	0.000	5.5	3687
P10	pH	W02	pHW2	0.000	6	3899
F01	Fa	W02	FaW2	2.605	2	1939
F02	Fa	W02	FaW2	2.416	2	1208
F03	Fa	W02	FaW2	0.000	2	1740
F04	Fa	W02	FaW2	2.721	0.5	806
F05	Fa	W02	FaW2	2.346	0	889
F06	Fa	W02	FaW2	3.303	1.5	825
F07	Fa	W02	FaW2	0.000	1.5	1332
F08	Fa	W02	FaW2	2.264	1.5	1329
F09	Fa	W02	FaW2	1.533	1	676
F10	Fa	W02	FaW2	0.000	0.5	1023
H01	He	W02	HeW2	2.612	1	936
H02	He	W02	HeW2	0.000	3	3836
H03	He	W02	HeW2	1.435	2	2736
H04	He	W02	HeW2	2.722	2	2964
H05	He	W02	HeW2	2.354	3	3715
H06	He	W02	HeW2	0.000	4	3769
H07	He	W02	HeW2	2.426	2	3945
H08	He	W02	HeW2	3.017	2	3547
H09	He	W02	HeW2	1.539	0.5	2400
H10	He	W02	HeW2	0.000	4	3762
N01	Nu	W05	NuW5	3.601	2	3308
N02	Nu	W05	NuW5	0.000	3	3474
N03	Nu	W05	NuW5	3.410	3	3403
N04	Nu	W05	NuW5	2.740	3	3297
N05	Nu	W05	NuW5	4.016	2.5	2853

N06	Nu	W05	NuW5	3.046	2	1990
N07	Nu	W05	NuW5	3.260	2	2955
N08	Nu	W05	NuW5	3.228	3	3251
N09	Nu	W05	NuW5	2.509	2	3177
N10	Nu	W05	NuW5	3.755	2	2421
P01	pH	W05	pHW5	1.889	2	2487
P02	pH	W05	pHW5	3.103	1	2086
P03	pH	W05	pHW5	3.020	3.5	2810
P04	pH	W05	pHW5	3.549	2	2029
P05	pH	W05	pHW5	3.393	2.5	3056
P06	pH	W05	pHW5	2.108	2	1957
P07	pH	W05	pHW5	3.082	2	2752
P08	pH	W05	pHW5	3.123	4	3001
P09	pH	W05	pHW5	2.823	3	3560
P10	pH	W05	pHW5	3.070	3	2940
F01	Fa	W05	FaW5	1.250	3	3541
F02	Fa	W05	FaW5	0.000	3.5	3570
F03	Fa	W05	FaW5	3.186	3	3718
F04	Fa	W05	FaW5	1.290	0	3570
F05	Fa	W05	FaW5	1.302	0	3537
F06	Fa	W05	FaW5	1.672	3.5	3498
F07	Fa	W05	FaW5	1.735	3	3337
F08	Fa	W05	FaW5	0.798	2	1981
F09	Fa	W05	FaW5	1.880	3.5	3569
F10	Fa	W05	FaW5	2.377	2	1927
H01	He	W05	HeW5	2.574	3	4198
H02	He	W05	HeW5	0.000	2	3890
H03	He	W05	HeW5	0.000	4	3867
H04	He	W05	HeW5	3.896	2	3695
H05	He	W05	HeW5	3.060	3.5	3658
H06	He	W05	HeW5	2.275	1	2258
H07	He	W05	HeW5	3.955	1.5	2515
H08	He	W05	HeW5	3.629	3	3512
H09	He	W05	HeW5	3.629	2	3172
H10	He	W05	HeW5	4.081	2	2484
N01	Nu	W08	NuW8	3.023	4	3995
N02	Nu	W08	NuW8	0.000	3.5	3864
N03	Nu	W08	NuW8	4.196	3	4286
N04	Nu	W08	NuW8	3.300	4	3770
N05	Nu	W08	NuW8	3.246	3	3897
N06	Nu	W08	NuW8	2.599	2	3605
N07	Nu	W08	NuW8	2.907	2.5	3855
N08	Nu	W08	NuW8	3.092	2.5	3697
N09	Nu	W08	NuW8	3.058	2.5	3815
N10	Nu	W08	NuW8	3.502	4	3685
P01	pH	W08	pHW8	0.000	3	3691

P02	pH	W08	pHW8	0.000	4	4031
P03	pH	W08	pHW8	3.295	3.5	3691
P04	pH	W08	pHW8	3.342	3.5	4508
P05	pH	W08	pHW8	3.177	4.5	3657
P06	pH	W08	pHW8	0.000	2.5	3515
P07	pH	W08	pHW8	3.162	3	3450
P08	pH	W08	pHW8	3.794	4.5	4144
P09	pH	W08	pHW8	2.706	5	5380
P10	pH	W08	pHW8	3.206	4.5	3873
F01	Fa	W08	FaW8	2.925	3.5	4021
F02	Fa	W08	FaW8	0.000	3.5	3982
F03	Fa	W08	FaW8	3.138	4	4090
F04	Fa	W08	FaW8	2.780	3.5	3751
F05	Fa	W08	FaW8	2.115	3.5	3691
F06	Fa	W08	FaW8	2.624	4	3921
F07	Fa	W08	FaW8	2.920	3	3756
F08	Fa	W08	FaW8	1.600	2.5	3923
F09	Fa	W08	FaW8	3.133	4	3711
F10	Fa	W08	FaW8	3.060	3	3927
H01	He	W08	HeW8	2.984	3.5	4072
H02	He	W08	HeW8	0.000	3.5	4196
H03	He	W08	HeW8	2.846	4.5	4259
H04	He	W08	HeW8	2.177	4	4367
H05	He	W08	HeW8	2.690	4.5	3974
H06	He	W08	HeW8	2.973	3.5	3786
H07	He	W08	HeW8	1.714	4	3748
H08	He	W08	HeW8	2.894	4.5	4142
H09	He	W08	HeW8	3.177	4	3934
H10	He	W08	HeW8	3.103	4	5380
N01	Nu	W11	NuW11	2.734	6.5	6513
N02	Nu	W11	NuW11	2.281	6.5	6841
N03	Nu	W11	NuW11	2.979	5.5	6716
N04	Nu	W11	NuW11	2.800	6	7107
N05	Nu	W11	NuW11	3.444	4.5	6469
N06	Nu	W11	NuW11	2.686	4.5	5139
N07	Nu	W11	NuW11	2.714	6	6869
N08	Nu	W11	NuW11	2.345	5	6509
N09	Nu	W11	NuW11	2.591	4.5	5948
N10	Nu	W11	NuW11	3.129	6.5	6683
P01	pH	W11	pHW11	2.697	5	5480
P02	pH	W11	pHW11	2.281	6.5	6568
P03	pH	W11	pHW11	2.979	6	6575
P04	pH	W11	pHW11	2.800	7	7087
P05	pH	W11	pHW11	3.444	6.5	6858
P06	pH	W11	pHW11	2.686	4	3200
P07	pH	W11	pHW11	2.714	5	4336

P08	pH	W11	pHW11	2.426	8	7381
P09	pH	W11	pHW11	2.492	7	6896
P10	pH	W11	pHW11	3.129	5.5	6286
F01	Fa	W11	FaW11	2.954	6	6018
F02	Fa	W11	FaW11	1.844	5	6147
F03	Fa	W11	FaW11	2.236	5.5	6388
F04	Fa	W11	FaW11	2.676	6	5853
F05	Fa	W11	FaW11	2.673	5.5	5794
F06	Fa	W11	FaW11	2.945	7	6501
F07	Fa	W11	FaW11	4.169	5	5267
F08	Fa	W11	FaW11	0.000	4.5	5306
F09	Fa	W11	FaW11	2.747	6.5	7184
F10	Fa	W11	FaW11	0.000	4	4329
H01	He	W11	HeW11	0.000	5	7002
H02	He	W11	HeW11	0.000	5	5415
H03	He	W11	HeW11	2.566	6	7181
H04	He	W11	HeW11	0.000	5	6044
H05	He	W11	HeW11	4.592	6	7255
H06	He	W11	HeW11	3.360	5	7009
H07	He	W11	HeW11	2.997	5.5	7097
H08	He	W11	HeW11	0.000	5.5	6186
H09	He	W11	HeW11	3.336	6.5	6287
H10	He	W11	HeW11	0.000	6	7203
N01	Nu	W14	NuW14	0.000		
N02	Nu	W14	NuW14	2.877		
N03	Nu	W14	NuW14	3.193		
N04	Nu	W14	NuW14	3.120		
N05	Nu	W14	NuW14	4.432		
N06	Nu	W14	NuW14	2.931		
N07	Nu	W14	NuW14	3.027		
N08	Nu	W14	NuW14	2.783		
N09	Nu	W14	NuW14	2.889		
N10	Nu	W14	NuW14	3.301		
P01	pH	W14	pHW14	0.000		
P02	pH	W14	pHW14	0.000		
P03	pH	W14	pHW14	2.985		
P04	pH	W14	pHW14	2.894		
P05	pH	W14	pHW14	3.567		
P06	pH	W14	pHW14	0.000		
P07	pH	W14	pHW14	2.569		
P08	pH	W14	pHW14	2.349		
P09	pH	W14	pHW14	2.610		
P10	pH	W14	pHW14	2.373		
F01	Fa	W14	FaW14	2.676		
F02	Fa	W14	FaW14	2.127		
F03	Fa	W14	FaW14	2.841		

F04	Fa	W14	FaW14	2.875		
F05	Fa	W14	FaW14	0.000		
F06	Fa	W14	FaW14	2.915		
F07	Fa	W14	FaW14	3.426		
F08	Fa	W14	FaW14	1.958		
F09	Fa	W14	FaW14	2.557		
F10	Fa	W14	FaW14	0.000		
H01	He	W14	HeW14	2.312		
H02	He	W14	HeW14	1.998		
H03	He	W14	HeW14	2.287		
H04	He	W14	HeW14	0.000		
H05	He	W14	HeW14	0.000		
H06	He	W14	HeW14	2.804		
H07	He	W14	HeW14	2.516		
H08	He	W14	HeW14	0.000		
H09	He	W14	HeW14	3.408		
H10	He	W14	HeW14	2.968		
N01	Nu	W17	NuW17	0.000	5	8394
N02	Nu	W17	NuW17	3.329	6	7309
N03	Nu	W17	NuW17	0.000	5	8471
N04	Nu	W17	NuW17	3.595	7	9479
N05	Nu	W17	NuW17	2.218	4.5	7968
N06	Nu	W17	NuW17	3.313	4.5	8568
N07	Nu	W17	NuW17	2.690	5.5	11057
N08	Nu	W17	NuW17	2.885	4.5	9918
N09	Nu	W17	NuW17	2.703	5	9357
N10	Nu	W17	NuW17	3.089	5	7371
P01	pH	W17	pHW17	2.656	5	8357
P02	pH	W17	pHW17	0.000	6	8755
P03	pH	W17	pHW17	0.000	7	11291
P04	pH	W17	pHW17	2.673	7	12809
P05	pH	W17	pHW17	2.862	6	7359
P06	pH	W17	pHW17	0.000	4.5	6792
P07	pH	W17	pHW17	2.813	5.5	6395
P08	pH	W17	pHW17	3.040	7.5	9545
P09	pH	W17	pHW17	2.715	6.5	11371
P10	pH	W17	pHW17	2.871	6	10179
F01	Fa	W17	FaW17	0.000	5	4101
F02	Fa	W17	FaW17	0.000	5	7078
F03	Fa	W17	FaW17	2.888	4.5	7633
F04	Fa	W17	FaW17	2.960	4	6039
F05	Fa	W17	FaW17	2.833	5	6608
F06	Fa	W17	FaW17	3.502	5	5642
F07	Fa	W17	FaW17	3.510	4	4461
F08	Fa	W17	FaW17	0.000	3	4040
F09	Fa	W17	FaW17	2.742	5	6489

F10	Fa	W17	FaW17	0.000	3	2760
H01	He	W17	HeW17	0.000	5.5	6953
H02	He	W17	HeW17	0.000	4.5	4334
H03	He	W17	HeW17	0.000	5.5	8421
H04	He	W17	HeW17	2.621	4.5	5389
H05	He	W17	HeW17	0.000	6.5	7987
H06	He	W17	HeW17	0.000	5.5	7462
H07	He	W17	HeW17	0.000	5.5	8792
H08	He	W17	HeW17	0.000	6	5333
H09	He	W17	HeW17	3.818	6.5	8196
H10	He	W17	HeW17	2.328	5.5	9929
N01	Nu	W20	NuW20	0.000		
N02	Nu	W20	NuW20	2.770		
N03	Nu	W20	NuW20	0.000		
N04	Nu	W20	NuW20	3.326		
N05	Nu	W20	NuW20	2.354		
N06	Nu	W20	NuW20	3.549		
N07	Nu	W20	NuW20	3.061		
N08	Nu	W20	NuW20	3.198		
N09	Nu	W20	NuW20	3.071		
N10	Nu	W20	NuW20	2.795		
P01	pH	W20	pHW20	2.853		
P02	pH	W20	pHW20	0.000		
P03	pH	W20	pHW20	2.819		
P04	pH	W20	pHW20	2.987		
P05	pH	W20	pHW20	3.309		
P06	pH	W20	pHW20	0.000		
P07	pH	W20	pHW20	2.977		
P08	pH	W20	pHW20	3.037		
P09	pH	W20	pHW20	2.953		
P10	pH	W20	pHW20	2.598		
F01	Fa	W20	FaW20	3.099		
F02	Fa	W20	FaW20	2.419		
F03	Fa	W20	FaW20	2.657		
F04	Fa	W20	FaW20	3.493		
F05	Fa	W20	FaW20	2.812		
F06	Fa	W20	FaW20	3.022		
F07	Fa	W20	FaW20	3.552		
F08	Fa	W20	FaW20	3.616		
F09	Fa	W20	FaW20	2.689		
F10	Fa	W20	FaW20	0.000		
H01	He	W20	HeW20	0.000		
H02	He	W20	HeW20	0.000		
H03	He	W20	HeW20	0.000		
H04	He	W20	HeW20	0.000		
H05	He	W20	HeW20	2.221		

H06	He	W20	HeW20	2.957		
H07	He	W20	HeW20	2.848		
H08	He	W20	HeW20	0.000		
H09	He	W20	HeW20	3.443		
H10	He	W20	HeW20	2.835		
N01	Nu	W24	NuW24	2.792	4.5	4221
N02	Nu	W24	NuW24	3.508	4.5	2107
N03	Nu	W24	NuW24	0.000	4	2029
N04	Nu	W24	NuW24	3.973	4	4390
N05	Nu	W24	NuW24	2.869	3	2399
N06	Nu	W24	NuW24	4.146	3.5	3534
N07	Nu	W24	NuW24	3.947	5	11297
N08	Nu	W24	NuW24	3.540	4.5	4780
N09	Nu	W24	NuW24	3.684	3.5	3327
N10	Nu	W24	NuW24	3.680	3.5	1276
P01	pH	W24	pHW24	0.000	5	3991
P02	pH	W24	pHW24	0.000	6.5	6455
P03	pH	W24	pHW24	2.823	5	6689
P04	pH	W24	pHW24	3.020	6.5	10722
P05	pH	W24	pHW24	2.987	4.5	4988
P06	pH	W24	pHW24	2.685	6	6350
P07	pH	W24	pHW24	3.204	5.5	4007
P08	pH	W24	pHW24	2.308	6	5858
P09	pH	W24	pHW24	3.047	7	11080
P10	pH	W24	pHW24	2.353	5	4013
F01	Fa	W24	FaW24	2.974	4.5	7106
F02	Fa	W24	FaW24	NA	4	3902
F03	Fa	W24	FaW24	3.565	4	4306
F04	Fa	W24	FaW24	3.129	3	1395
F05	Fa	W24	FaW24	3.399	4.5	7793
F06	Fa	W24	FaW24	2.926	3.5	2525
F07	Fa	W24	FaW24	3.136	3.5	2722
F08	Fa	W24	FaW24	3.390	2	1245
F09	Fa	W24	FaW24	2.785	4.5	9788
F10	Fa	W24	FaW24	0.000	2	429
H01	He	W24	HeW24	2.819	4	7591
H02	He	W24	HeW24	0.000	4	4500
H03	He	W24	HeW24	0.000	4	4089
H04	He	W24	HeW24	0.000	3.5	3830
H05	He	W24	HeW24	3.175	5	11720
H06	He	W24	HeW24	3.200	4.5	9513
H07	He	W24	HeW24	NA	4.5	5528
H08	He	W24	HeW24	2.425	4	3493
H09	He	W24	HeW24	3.237	5	9412
H10	He	W24	HeW24	2.680	4.5	7379
N01	Nu	W26	NuW26	0.000		

N02	Nu	W26	NuW26	3.231		
N03	Nu	W26	NuW26	0.000		
N04	Nu	W26	NuW26	3.216		
N05	Nu	W26	NuW26	0.000		
N06	Nu	W26	NuW26	3.689		
N07	Nu	W26	NuW26	2.971		
N08	Nu	W26	NuW26	3.093		
N09	Nu	W26	NuW26	3.802		
N10	Nu	W26	NuW26	3.399		
P01	pH	W26	pHW26	0.000		
P02	pH	W26	pHW26	0.000		
P03	pH	W26	pHW26	2.617		
P04	pH	W26	pHW26	NA		
P05	pH	W26	pHW26	3.716		
P06	pH	W26	pHW26	NA		
P07	pH	W26	pHW26	3.446		
P08	pH	W26	pHW26	2.585		
P09	pH	W26	pHW26	3.149		
P10	pH	W26	pHW26	2.735		
F01	Fa	W26	FaW26	3.415		
F02	Fa	W26	FaW26	NA		
F03	Fa	W26	FaW26	3.732		
F04	Fa	W26	FaW26	3.237		
F05	Fa	W26	FaW26	3.059		
F06	Fa	W26	FaW26	3.059		
F07	Fa	W26	FaW26	3.156		
F08	Fa	W26	FaW26	2.891		
F09	Fa	W26	FaW26	NA		
F10	Fa	W26	FaW26	0.000		
H01	He	W26	HeW26	3.055		
H02	He	W26	HeW26	0.000		
H03	He	W26	HeW26	0.000		
H04	He	W26	HeW26	0.000		
H05	He	W26	HeW26	2.925		
H06	He	W26	HeW26	0.000		
H07	He	W26	HeW26	NA		
H08	He	W26	HeW26	0.000		
H09	He	W26	HeW26	3.041		
H10	He	W26	HeW26	2.438		
N01	Nu	W29	NuW29	0.000	5	5540
N02	Nu	W29	NuW29	3.660	5	4049
N03	Nu	W29	NuW29	0.000	4	3642
N04	Nu	W29	NuW29	3.035	6	10695
N05	Nu	W29	NuW29	2.201	3.5	2208
N06	Nu	W29	NuW29	3.601	4	6623
N07	Nu	W29	NuW29	3.392	5.5	13795

N08	Nu	W29	NuW29	3.640	5	7450
N09	Nu	W29	NuW29	2.723	5	6847
N10	Nu	W29	NuW29	2.927	4	2267
P01	pH	W29	pHW29	2.446	4.5	2316
P02	pH	W29	pHW29	NA	5.5	7053
P03	pH	W29	pHW29	2.589	5.5	6188
P04	pH	W29	pHW29	NA	6.5	10418
P05	pH	W29	pHW29	2.766	4.5	5047
P06	pH	W29	pHW29	NA	4.5	5010
P07	pH	W29	pHW29	2.815	4.5	1932
P08	pH	W29	pHW29	2.753	5	5454
P09	pH	W29	pHW29	2.678	6	9446
P10	pH	W29	pHW29	2.905	4	2815
F01	Fa	W29	FaW29	2.711	4.5	6598
F02	Fa	W29	FaW29	2.570	4	6510
F03	Fa	W29	FaW29	2.923	4	4828
F04	Fa	W29	FaW29	NA	4	2869
F05	Fa	W29	FaW29	3.109	4.5	4021
F06	Fa	W29	FaW29	3.161	4	3140
F07	Fa	W29	FaW29	3.388	3.5	3381
F08	Fa	W29	FaW29	2.735	3	1466
F09	Fa	W29	FaW29	NA	4.5	7414
F10	Fa	W29	FaW29	0.000	2	345
H01	He	W29	HeW29	3.061	5	8510
H02	He	W29	HeW29	0.000	4.5	3001
H03	He	W29	HeW29	0.000	4.5	4531
H04	He	W29	HeW29	0.000	4	3875
H05	He	W29	HeW29	2.265	6.5	11793
H06	He	W29	HeW29	2.322	6	15080
H07	He	W29	HeW29	2.663	5.5	5896
H08	He	W29	HeW29	0.000	4.5	2812
H09	He	W29	HeW29	2.676	6	9716
H10	He	W29	HeW29	2.201	4.5	11368
N01	Nu	W32	NuW32	0.000		
N02	Nu	W32	NuW32	3.871		
N03	Nu	W32	NuW32	0.000		
N04	Nu	W32	NuW32	4.243		
N05	Nu	W32	NuW32	2.905		
N06	Nu	W32	NuW32	4.296		
N07	Nu	W32	NuW32	NA		
N08	Nu	W32	NuW32	3.493		
N09	Nu	W32	NuW32	4.440		
N10	Nu	W32	NuW32	2.851		
P01	pH	W32	pHW32	NA		
P02	pH	W32	pHW32	NA		
P03	pH	W32	pHW32	NA		

P04	pH	W32	pHW32	NA		
P05	pH	W32	pHW32	0.000		
P06	pH	W32	pHW32	NA		
P07	pH	W32	pHW32	3.773		
P08	pH	W32	pHW32	NA		
P09	pH	W32	pHW32	2.775		
P10	pH	W32	pHW32	3.208		
F01	Fa	W32	FaW32	3.274		
F02	Fa	W32	FaW32	2.068		
F03	Fa	W32	FaW32	3.598		
F04	Fa	W32	FaW32	NA		
F05	Fa	W32	FaW32	3.029		
F06	Fa	W32	FaW32	3.599		
F07	Fa	W32	FaW32	3.116		
F08	Fa	W32	FaW32	2.942		
F09	Fa	W32	FaW32	NA		
F10	Fa	W32	FaW32	0.000		
H01	He	W32	HeW32	2.336		
H02	He	W32	HeW32	0.000		
H03	He	W32	HeW32	0.000		
H04	He	W32	HeW32	0.000		
H05	He	W32	HeW32	3.416		
H06	He	W32	HeW32	2.622		
H07	He	W32	HeW32	2.730		
H08	He	W32	HeW32	0.000		
H09	He	W32	HeW32	3.381		
H10	He	W32	HeW32	2.897		
N01	Nu	W35	NuW35	0.000	4.5	6188
N02	Nu	W35	NuW35	3.141	5	6395
N03	Nu	W35	NuW35	0.000	3.5	2131
N04	Nu	W35	NuW35	3.595	6	12336
N05	Nu	W35	NuW35	0.000	3	1990
N06	Nu	W35	NuW35	2.517	3.5	3830
N07	Nu	W35	NuW35	NA	6.5	16473
N08	Nu	W35	NuW35	0.000	5.5	8028
N09	Nu	W35	NuW35	2.100	5	7623
N10	Nu	W35	NuW35	NA	4.5	2465
P01	pH	W35	pHW35	NA	4.5	3530
P02	pH	W35	pHW35	NA	5	4756
P03	pH	W35	pHW35	NA	5	5553
P04	pH	W35	pHW35	NA	6.5	11315
P05	pH	W35	pHW35	3.413	5.5	7108
P06	pH	W35	pHW35	NA	4	2834
P07	pH	W35	pHW35	3.681	4	2372
P08	pH	W35	pHW35	NA	5	5612
P09	pH	W35	pHW35	2.474	7	13364

P10	pH	W35	pHW35	2.528	4.5	4294
F01	Fa	W35	FaW35	2.871	4.5	10567
F02	Fa	W35	FaW35	NA	4	6779
F03	Fa	W35	FaW35	2.876	4	4502
F04	Fa	W35	FaW35	2.168	3.5	2634
F05	Fa	W35	FaW35	2.850	4	5819
F06	Fa	W35	FaW35	3.212	4	3349
F07	Fa	W35	FaW35	3.231	4	3984
F08	Fa	W35	FaW35	3.082	3	1982
F09	Fa	W35	FaW35	0.000	4	8010
F10	Fa	W35	FaW35	0.000	2	445
H01	He	W35	HeW35	2.732	5	8762
H02	He	W35	HeW35	0.000	3.5	2062
H03	He	W35	HeW35	0.000	5	4376
H04	He	W35	HeW35	0.000	4	3457
H05	He	W35	HeW35	2.582	6.5	10079
H06	He	W35	HeW35	2.139	6.5	13518
H07	He	W35	HeW35	3.142	5	8467
H08	He	W35	HeW35	0.000	4	2028
H09	He	W35	HeW35	2.557	6.5	12563
H10	He	W35	HeW35	2.192	4.5	11673
N01	Nu	W38	NuW38	0.000		
N02	Nu	W38	NuW38	3.804		
N03	Nu	W38	NuW38	0.000		
N04	Nu	W38	NuW38	3.968		
N05	Nu	W38	NuW38	2.182		
N06	Nu	W38	NuW38	3.700		
N07	Nu	W38	NuW38	2.695		
N08	Nu	W38	NuW38	NA		
N09	Nu	W38	NuW38	3.502		
N10	Nu	W38	NuW38	NA		
P01	pH	W38	pHW38	NA		
P02	pH	W38	pHW38	NA		
P03	pH	W38	pHW38	2.521		
P04	pH	W38	pHW38	0.000		
P05	pH	W38	pHW38	NA		
P06	pH	W38	pHW38	1.760		
P07	pH	W38	pHW38	3.565		
P08	pH	W38	pHW38	3.409		
P09	pH	W38	pHW38	0.000		
P10	pH	W38	pHW38	2.704		
F01	Fa	W38	FaW38	3.154		
F02	Fa	W38	FaW38	NA		
F03	Fa	W38	FaW38	3.742		
F04	Fa	W38	FaW38	2.201		
F05	Fa	W38	FaW38	3.439		

F06	Fa	W38	FaW38	3.625		
F07	Fa	W38	FaW38	3.438		
F08	Fa	W38	FaW38	3.129		
F09	Fa	W38	FaW38	2.653		
F10	Fa	W38	FaW38	0.000		
H01	He	W38	HeW38	2.936		
H02	He	W38	HeW38	0.000		
H03	He	W38	HeW38	0.000		
H04	He	W38	HeW38	0.000		
H05	He	W38	HeW38	3.701		
H06	He	W38	HeW38	NA		
H07	He	W38	HeW38	2.893		
H08	He	W38	HeW38	0.000		
H09	He	W38	HeW38	3.403		
H10	He	W38	HeW38	2.804		
N01	Nu	W41	NuW41	0.000	4.5	7193
N02	Nu	W41	NuW41	3.716	5.5	11257
N03	Nu	W41	NuW41	0.000	4	3168
N04	Nu	W41	NuW41	4.322	5.5	13167
N05	Nu	W41	NuW41	2.481	3.5	2239
N06	Nu	W41	NuW41	3.447	3.5	7322
N07	Nu	W41	NuW41	3.262	5.5	14011
N08	Nu	W41	NuW41	NA	4	6603
N09	Nu	W41	NuW41	3.727	5.5	8612
N10	Nu	W41	NuW41	NA	5.5	3986
P01	pH	W41	pHW41	0.000	4	3088
P02	pH	W41	pHW41	NA	5	5763
P03	pH	W41	pHW41	NA	4.5	6374
P04	pH	W41	pHW41	NA	6.5	7268
P05	pH	W41	pHW41	NA	5	5855
P06	pH	W41	pHW41	2.502	4	3408
P07	pH	W41	pHW41	3.970	4.5	2718
P08	pH	W41	pHW41	3.580	4.5	6586
P09	pH	W41	pHW41	2.980	6.5	11081
P10	pH	W41	pHW41	2.830	5	4382
F01	Fa	W41	FaW41	3.545	4.5	9046
F02	Fa	W41	FaW41	2.816	4	6653
F03	Fa	W41	FaW41	3.672	3.5	4059
F04	Fa	W41	FaW41	2.232	3	2781
F05	Fa	W41	FaW41	3.290	3.5	5767
F06	Fa	W41	FaW41	3.640	3.5	4574
F07	Fa	W41	FaW41	3.432	3.5	4505
F08	Fa	W41	FaW41	2.878	3	1939
F09	Fa	W41	FaW41	3.049	3.5	7792
F10	Fa	W41	FaW41	1.923	2	341
H01	He	W41	HeW41	3.390	4.5	13496

H02	He	W41	HeW41	0.000	4	1951
H03	He	W41	HeW41	0.000	4.5	4406
H04	He	W41	HeW41	0.000	4	4356
H05	He	W41	HeW41	3.598	6	9559
H06	He	W41	HeW41	NA	6.5	11602
H07	He	W41	HeW41	2.996	5.5	8530
H08	He	W41	HeW41	0.000	4	449
H09	He	W41	HeW41	3.841	6.5	8311
H10	He	W41	HeW41	3.465	5.5	12322
