# Supplementary material for: Chickens can durably clear herpesvirus vaccine infection in feathers while still carrying vaccine-induced antibodies
Source: Vet Res. 2020 Feb 24;51:24. doi: 10.1186/s13567-020-00749-1 (PMC7041111; doi:10.1186/s13567-020-00749-1)
Supplement: Supplementary file 2 — Additional file 2. HVT loads in spleens at week 41. [file 13567_2020_749_MOESM2_ESM.pdf]

Additional file 2. HVT loads in spleens at week 41

| Bird | HVT load (log) |
|---|---|
| N1 | NI* |
| N2 | 4,813 |
| N3 | 0,000 |
| N4 | 3,927 |
| N5 | 1,728 |
| N6 | 3,907 |
| N7 | 3,846 |
| N8 | 3,562 |
| N9 | 3,932 |
| N10 | 3,572 |
| P1 | 1,806 |
| P2 | 3,628 |
| P3 | 4,440 |
| P4 | 3,318 |
| P5 | 3,923 |
| P6 | 2,378 |
| P7 | 3,466 |
| P8 | 3,248 |
| P9 | 2,939 |
| P10 | 3,992 |
| F1 | 3,758 |
| F2 | 4,619 |
| F3 | 4,056 |
| F4 | 3,081 |
| F5 | 4,132 |
| F6 | 3,872 |
| F7 | 4,001 |
| F8 | 4,118 |
| F9 | 2,981 |
| F10 | 1,966 |
| H1 | 2,987 |
| H2 | 0,000 |
| H3 | 0,000 |
| H4 | 0,000 |
| H5 | 3,310 |
| H6 | 3,772 |
| H7 | 2,857 |
| H8 | no DNA |
| H9 | 3,745 |
| H10 | NI |

* NI: uninterpretable
